# A study on the long-term efficacy of Seresto® collars in preventing *Babesia canis* (Piana & Galli-Valerio, 1895) transmission to dogs by infected *Dermacentor reticulatus* (Fabricius, 1794) ticks

**DOI:** 10.1186/s13071-019-3393-z

**Published:** 2019-03-22

**Authors:** Josephus J. Fourie, Christa de Vos, Dionne Crafford, Matthias Pollmeier, Bettina Schunack

**Affiliations:** 1 Clinvet International (Pty) Ltd, Uitzich Road, Bainsvlei, Bloemfontein, South Africa; 2 Bayer Animal Health GmbH, Monheim, Germany

**Keywords:** Seresto®, Imidacloprid, Flumethrin, *Babesia canis*, *Dermacentor reticulatus*, Transmission prevention

## Abstract

**Background:**

An imidacloprid/flumethrin collar (Seresto®) was previously shown to prevent infection with *Babesia canis*, transmitted by *Dermacentor reticulatus*, in dogs for up to 1 month after application. The present study evaluated the prevention of transmission throughout the claimed efficacy period of 8 months.

**Methods:**

Eight animals each were randomly included in groups 1 (negative control) and 2 (Seresto® collar), respectively. Animals in group 2 received the Seresto® collar on Day 0. Tick challenges were performed monthly from the 2nd to the 8th month. Assessment criteria included *in situ* tick counts 48 hours post-challenge, polymerase chain reaction (PCR) analyses and immunofluorescence assays (IFA). Whenever dogs were diagnosed with babesiosis they were “rescue-treated”, excluded and replaced. Consequently, 24 replacement animals were introduced at various time points throughout the study in the control group; thus data for a total of 32 dogs were available in the latter group at study termination.

**Results:**

Acaricidal efficacy for *in situ* counts was 93% on Day 30, and ranged from 97 to 100% thereafter. No *B. canis* specific DNA or antibodies were detected in any Seresto®-treated dog at any time. *Babesia canis*-specific DNA and antibodies were detected in 2–6 of 8 control dogs after each challenge, confirming the validity of the challenge model.

**Conclusions:**

The Seresto® collar was highly effective against challenges with *D. reticulatus* ticks for up to 8 months. The high sustained acaricidal efficacy over this period prevented transmission of *B. canis*, thus fully protecting dogs against infection in this experimental infestation model.

## Background

Worldwide, ticks transmit a broad range of diseases to dogs of which canine babesiosis is one of the most significant and clinically relevant. Clinical disease is caused by infections with both large and small forms of *Babesia* spp., with the species relevant to Europe being: *B. canis*, *B. vogeli*, *B. gibsoni*, and *B. microti*-like isolates also referred to as “*B. vulpes*” and “*Theileria annae*” [1]. The clinical signs, treatment and prognosis of infections with these *Babesia* spp. vary greatly, and the transmission and geographical distribution is mainly based on that of the competent tick vectors. In general, it is assumed that the least pathogenic large-sized species of *Babesia* is *B. vogeli*, and that the most virulent species in Europe is *B. canis*, transmitted mainly by the ornate dog tick, *Dermacentor reticulatus* [2]. This *Babesia* sp., like others in its genus, is able to invade ovaries of female *D. reticulatus* ticks and is transmitted transovarially to the next generation of larvae. Together with transstadial transmission, this feature results in *D. reticulatus* populations functioning as a reservoir, enabling maintenance of *B. canis* locally for several tick generations [3]. *Dermacentor reticulatus* is widely distributed over central Europe, but in recent years its distribution has considerably expanded in some regions. Large areas thought to be historically too cold for its survival and completion of its life-cycle, have now been invaded by this tick. Countries that have experienced a remarkable spread of this tick are Germany, Poland, Hungary and Slovakia, but also the Netherlands and Belgium, with the recent climatic changes being frequently reported as the predominant driving force [4, 5]. Other factors contributing to the spread of this tick are its wide host range that includes wild and domesticated mammals, and its extreme environmental survivability and tolerance. Adult ticks have been shown to survive for up to four years without a blood meal, tolerate extreme cold (−10 °C for 150 days under laboratory conditions), and even when submerged in cool clean water adult ticks survived for more than 100 days [6–8]. For the European market, a vaccine for dogs against *B. canis* is available, based on soluble parasite antigens that induce a partial protection for dogs newly exposed to *B. canis*. Vaccination does not prevent infection, but both shortens and diminishes the severity of their clinical signs; moreover, a lower parasitemia may result. Vaccination can be started from five months of age and requires annual re-vaccination, but does not cross-protect against other *Babesia* spp. The protection of dogs against infection with *B. canis* therefore primarily relies on the effective on-host control of the tick vector, thereby also reducing the tick population in the environment capable of transmitting infections.

To effectively prevent *B. canis* infections, rapid killing of ticks on the dog is required. As such, ticks need to be killed before maturation of the *B. canis* sporozoites present in their salivary glands, which has been reported to take up to 48 hours after attachment [9].

Once a *D. reticulatus* tick has taken a blood meal and feeding is interrupted, transmission time is shortened and was shown to have occurred within eight hours as demonstrated for male *D. reticulatus* [10]. The development of challenge models with unfed infected laboratory bred ticks made it possible to assess the efficacy of acaricidal products to prevent transmission of *B. canis*, which has been demonstrated for several treatments such as topically applied contact acaricides [11–13], and more recently systemic isoxazoline compounds [14–16]. Effective protection of dogs against infection not only relies on an immediate rapid killing effect, but also on sustaining this rapid killing effect over the entire period during which ticks are active in the environment, irrespective of a dog’s lifestyle (e.g. frequent swimming). Acaricidal collars with long-lasting sustained efficacy have been widely used against ticks on companion animals and may be ideally suited to protect dogs against *B. canis* infection, if the rapid killing effect can be sustained over these long periods of efficacy [17–19]. The rapid killing effect of the Seresto® collar (imidacloprid 10%/flumethrin 4.5%), with a proven sustained high level of efficacy against ticks [20], has previously been shown to effectively prevent infection with *B. canis* after a single challenge one month after collar application [11]. Consequently, this collar may have the potential to prevent the transmission of *B. canis* by infected ticks over the entire claimed efficacy period of eight months, making it an ideal candidate for protecting dogs against canine babesiosis over the entire tick activity season. The objective of the study was to determine the efficacy of the Seresto® collar in the prevention of transmission of *B. canis* by infected *D. reticulatus* ticks to dogs, for a time period consistent with the claimed efficacy period of eight months.

## Methods

### General design

The study was designed as a parallel group, randomised, single centre, negative controlled, efficacy study, and was conducted in compliance with the VICH GL9 on Good Clinical Practice and was approved by an institutional animal care and use committee (Clinvet IACUC). The study employed two groups, non-treated control group 1 and Seresto®-treated group 2, each consisting of 8 dogs. All dogs were acclimatized to the cage environment for at least 7 days before inclusion and random allocation to their study groups was based on body weight. Dogs in group 2 were fitted with the Seresto® collar on Day 0 and all dogs (groups 1 and 2) were subsequently challenged monthly with ticks starting on Day 28. Dogs were observed daily for general health. In addition, monthly tick counts, blood sampling and frequent veterinary examinations were performed (see activity schedule in Table 1).

**Table 1:** Activity schedule

Tick challenges	Tick counts	Blood sampling	Clinical examinations
Days 28, 56, 84, 112, 140, 168, 196 and 224	*In situ* counts on Days 30, 58, 86, 114, 142, 170, 198 and 226. Removal count on Day 231	IFA: Prior to inclusion and on Days-7, 28, 56, 84, 112, 140, 168, 196, 224 and 252; PCR: Following positive diagnosis on blood smears, as well as Day 252 for dogs where no infection was observed thus far	Day-7 and weekly thereafter up to Day 252

### Animals

Purpose-bred dogs, identified using electronic transponders with unique alphanumeric codes and belonging to Clinvet International (Pty) Ltd, were used in the study. At the time of enrolment all dogs were between 6 months and 6 years of age, and weighed between 10.2 and 31.6 kg. Dogs were ranked within sex by body weight. Within blocks of two, dogs were randomly allocated to the respective study groups. At baseline there was no statistically significant difference between groups in terms of hair length (ANOVA: *F*_(1,14)_ = 0.61, *P* = 0.4480), body weight (ANOVA: *F*_(1,14)_ = 0.00, *P* = 0.9719) and age (ANOVA: *F*_(1,14)_ = 0.28, *P* = 0.6078). In the control group a total of 32 animals were used over the duration of the study, whilst 9 animals in the Seresto® group were used in efficacy calculations. The control group consisted of 16 males and 16 females and the Seresto® group of 5 males and 4 females. All dogs were healthy (based on an examination by a veterinarian) and seronegative for *B. canis* antibodies prior to inclusion in the study. Dogs were individually housed in indoor pens, were fed a commercial dog food once daily and provided water *ad libitum*.

### Tick challenges and tick counting procedures

A laboratory-bred *B. canis*-infected *D. reticulatus* tick strain was used in the artificial infestations. The ticks used for artificial infestations originated from a single batch of ticks infected with *B. canis* as described by Jongejan et al. [12]. The infection rate of the tick batch used for artificial infestations was 8%, and was determined by PCR testing of a randomly selected sample of 50 ticks taken from the specific tick batch. Tick challenges were performed by releasing 50 unfed ticks with an equal gender distribution in an infestation crate, placing the dog in the crate and subsequently exposing the dog to the ticks for approximately 1 h (after which the dog was removed from the crate and the crate with remaining ticks, if any, removed from the pen). *In situ* tick counts were performed approximately 48 h after each tick challenge by direct observation following parting of the hair coat and palpation. A final removal count for all dogs was performed on Day 231 (7 days after the last tick challenge).

### Clinical examinations

All dogs were observed daily for general health and examined weekly by a veterinarian. Additional clinical examinations were conducted by a veterinarian on dogs displaying clinical signs [for example elevated body temperature (> 39.4 °C), listlessness, inappetence, anaemia, haematuria and/or icterus] associated with babesiosis. Rectal body temperatures were recorded three times per week from Days 28 to 252.

### Laboratory examinations

Serum was collected for immunofluorescence assays (IFA) from all dogs prior to inclusion in the study (Day -7) and monthly thereafter starting on Day 28 (i.e. Days 28, 56, 84, 112, 140, 168, 196, 224 and 252), and assayed for *B. canis* antibodies using a commercial test kit (MegaFLUO® BABESIA canis, Interlab, Vilnius, Lithuania). Serum was diluted 1:160 for screening and samples at this dilution exhibiting a positive fluorescence pattern similar to the positive control, were considered positive. Cross-reactivity with related organisms is not reported by either the manufacturer or in published literature.

Additionally, blood smears were prepared and examined for all dogs displaying signs of babesiosis [which included elevated body temperature (> 39.4 °C) and signs such as listlessness and inappetence], and whole blood samples for polymerase chain reaction (PCR) analysis were collected if infections were observed (Table 2).

**Table 2:** Diagnostic summary (blood smears, PCR and IFA)

Animal ID	First tick challenge day	Last tick challenge day	Total no. of challenges	*B. canis* detected on blood smear (Day)	*B. canis* DNA detected by PCR (Day)	Antibodies detected by IFA (Day)^b^
4EF 726	28	56	2	70	70	84
4F2 719	28	56	2	66	66	84
CC2 D5A	28	56	2	68	68	84
DF4 FD4	28	56	2	65	65	84
DF6 939	28	84	3	93	93	112
DF6 AE1	28	28	1	42	39	56
DF7 98F	28	112	4	128	128	140
E19 4E8	28	28	1	37	37	56
B29 DBD	56	56	1	64	64	84
28D 55B	84	224	6	nd	nd^a^	nd
5A1 364	84	84	1	93	93	112
5A6 66D	84	84	1	93	93	112
698 469	84	84	1	92	92	112
CC2 6AC	84	84	1	93	93	112
E18 24C	56	84	1	95	95	112
5A3 C4C	112	112	1	121	121	140
5A6 952	112	112	1	121	121	140
5A8 170	112	112	1	123	123	140
5D3 CA0	112	224	5	nd	nd^a^	nd
689 702	112	112	1	121	121	140
698 04E	112	168	3	177	177	196
697 F9D	140	168	2	176	176	196
698 1A0	140	224	4	233	233	252
698 22F	140	224	4	nd	nd^a^	nd
6D3 CE2	140	140	1	148	148	168
6D6 412	140	140	1	148	148	168
57C 00E	168	196	2	212	212	224
5A4 2FD	168	196	2	216	202	196
2A6 EA8	196	224	2	nd	nd^a^	nd
2AD 0E4	196	224	2	nd	nd^a^	nd
5BC D82	224	224	1	nd	nd^a^	nd
B2F AAB	224	224	1	233	233	252
2A8 C98	28	224	8	nd	nd^a^	nd
2AB E8B	28	224	8	nd	nd^a^	nd
B2B 68D	28	224	8	–	nd^a^	nd
CC3 20D	28	224	8	nd	nd^a^	nd
DF5 A66	28	224	8	nd	nd^a^	nd
DF7 5C1	28	224	8	nd	nd^a^	nd
E17 329	28	224	8	nd	nd^a^	nd
EA0 7C7	28	84	3	nd	–	nd
5A1 979	112	224	5	–	nd^a^	nd

^a^Blood collected and PCR performed at the end of the study assessment period

^b^IFA testing performed on Days-7, 28, 56, 84, 112, 140, 168, 196, 224 and 252. Results only show Days where antibodies were detected or indicated “nd” where no antibodies were detected during any of the sequential tests

*Abbreviations*: nd, not detected; –, not evaluated

On the final assessment day (Day 252), blood was collected from all dogs where no infection was observed up to that point and submitted for PCR analysis. This was done to ensure that these dogs indeed remained uninfected. Total genomic DNA was isolated from these samples using a commercial genomic DNA isolation kit (GeneJET Genomic DNA Purification Kit, Thermo Fisher Scientific, Waltham, MA, USA). Primers specific to the *B. canis* ITS region were used [14]. PCRs were performed using 20 µl blood with Phire Green HotStart II PCR Master Mix (Thermo Fisher Scientific) containing 500 nM of each primer *Babesia* 2F (5′-GGA AGG AGA AGT CGT AAC AAG GTT TCC-3′) and *B. canis* 2R (5′-CAG TGG TCA CAG ACC GGT CG-3′). Up to 400 ng DNA served as template, with an artificial plasmid (incorporating the specific primer sequences) that served as internal amplification control to minimize false negative results due to inhibition of the PCR. Thermal cycling entailed initial denaturation at 98 °C for 5 min followed by 45 cycles of 98 °C for 5 s, 68 °C for 5 s and 72 °C for 30 s, and was concluded with a final elongation step of 5 min at 72 °C. PCR detection sensitivities were performed using the target sequence, cloned into pSMART-HC Kan (Lucigen, Middleton, WI, USA) and sequence verified. The plasmid DNA was linearized using a restriction enzyme that digested the plasmid backbone, followed by DNA concentration determination using a NanoDrop 2000 (Thermo Fisher Scientific). Copy numbers were calculated and dilutions were prepared. The limit of detection was determined to be 5 copies per PCR.

A dog was regarded as infected with *B. canis* when positive for *B. canis* antibodies (IFA), positive for presence of *B. canis* by blood smear examination and confirmed by PCR.

### Rescue treatments

Once *Babesia* sp. organisms were detected in a blood smear the respective dog was rescue-treated with diminazene (Berenil® R.T.U., MSD Animal Heath, Kenilworth, NJ, USA) once-off, followed by a single administration of imidocarb dipropionate (Forray® 65, MSD Animal Health) 1 day later. Rescue-treated dogs were followed up until seroconversion was observed. To maintain the group size at 8 dogs per group for all tick challenges throughout the study, all dogs that became infected with *B. canis*, or in the treated group that lost or destroyed their collar, were replaced from a pool of non-treated and treated naïve animals already acclimatized to the study cage environment.

### Methods for calculating efficacy

#### Efficacy in preventing *Babesia canis* transmission

The percentage prevention of transmission efficacy against *B. canis* in the Seresto®-treated group was the primary efficacy criterion and calculated as follows:

$${\text{Efficacy }}\left( \% \right) \, = { 1}00 \, \times \, \left( {{\text{T}}_{\text{c }} {-}{\text{ T}}_{\text{t}} } \right)/{\text{T}}_{\text{c}}$$

where T_c_ is the total number of non-treated control dogs that became infected during the study; and T_t_ is the total number of Seresto®-treated dogs that became infected during the study.

#### Acaricidal efficacy

The acaricidal efficacy in the Seresto®–treated group at each assessment day was the secondary efficacy criterion and calculated according to the formula (based on geometric means):

$${\text{Efficacy }}\left( \% \right) \, = { 1}00 \, \times \, \left( {{\text{Gm}}_{\text{c }} {-}{\text{ Gm}}_{\text{t}} } \right)/{\text{Gm}}_{\text{c}}$$

where Gm_c_ is the geometric mean number of live ticks on dogs in the non-treated control group at a specific time point; and Gm_t_ is the geometric mean number of live ticks on dogs in the Seresto®-treated group at a specific time point.

The use of GMs is justified due to the challenge model and counting procedure employed.

### Statistical analysis

The statistical unit was the individual animal. Two different statistical analyses were used to assess the data. First an ANOVA with treatment effect was performed to estimate the acaricidal efficacy for each time point. Secondly a Fisher’s Exact test was used to compare the proportion of animals infected in each group. All analyses were two-sided with a level of significance of 5% and conducted in SAS Version 9.3 TS Level 1M2.

## Results

The Seresto® collars were well tolerated by all 9 treated dogs. One treated dog (EA0 7C7) destroyed its collar on Day 98 and was replaced (5A1 979) from the pool of treated naïve animals. In the control group a total of 32 (8 + 24) animals were used over the duration of the study. Out of these, 26 dogs were confirmed infected with *B. canis* based on blood smear evaluation over the entire study assessment period. These 26 control dogs (81.25%) also tested positive for *B. canis* infections on PCR and for *B. canis* specific antibodies on IFA. In the control group 2–6 of 8 dogs were infected after each challenge, confirming the validity of the challenge model. A number of dogs (1 Seresto®-treated and 19 controls) displayed elevated body temperatures (> 39.4 °C) on various occasions, mostly transient in nature. The majority of control dogs that became infected showed those elevated body temperatures around the time of confirmatory diagnosis, but there was no clear correlation with the presence of *B. canis* infection.

Overall, *B. canis* infections were detected by IFA and blood smear/PCR in 26 out of 32 (81.25%) non-treated dogs and in none of the Seresto®-treated dogs over the 252-day assessment period. The proportion of animals infected in each group differed statistically significantly (Fisher’s exact test, *P *< 0.0001; OR = 0.0144; 95% CI: 0.0007–0.2836) in favour of the Seresto®-treated group and the efficacy of the Seresto® collar in preventing transmission of *B. canis* was 100% over the challenge period of 224 days.

The arithmetic mean number of live ticks on the various assessment days for the two study groups are summarised in Table 3.

**Table 3:** Geometric mean tick count and acaricidal efficacy

Day	Geometric mean	Geometric mean (% efficacy)	*P*-value^a^
	Group 1	Group 2	
Day 30	5.8	0.4 (93.0)	*F*_(1,14)_ = 23.11, *P* = 0.0003
Day 58	4.1	0 (100)	*F*_(1,14)_ = 46.83, *P *< 0.0001
Day 86	4.9	0.1 (97.0)	*F*_(1,14)_ = 62.62, *P *< 0.0001
Day 114	8.6	0.1 (99.0)	*F*_(1,14)_ = 128.27, *P *< 0.0001
Day 142	3.1	0 (100)	*F*_(1,14)_ = 21.06, *P* = 0.0004
Day 170	4.4	0 (100)	*F*_(1,14)_ = 62.28, *P *< 0.0001
Day 198	8.7	0 (100)	*F*_(1,14)_ = 99.57, *P *< 0.0001
Day 226	5.4	0.1 (98.3)	*F*_(1,14)_ = 83.22, *P *< 0.0001
Day 231^b^	4.1	0.1 (97.8)	*F*_(1,14)_ = 21.48, *P* = 0.0004

^a^*P*-value: One-way ANOVA with a treatment effect presented as test statistic (*F*-value) plus degrees of freedom with exact *P*-value

^b^Removal count on Day 231, *in situ* count on all other assessment days

*Note*: Group 1: Non-treated control dogs; Group 2: Seresto®-treated dogs

The geometric mean number of live ticks recorded for the non-treated control group ranged from 3.1–8.7 for the duration of the study, and was significantly higher than that observed for the Seresto®-treated group. Based on geometric mean counts, the Seresto® collar was 93% effective on Day 30, and 97–100% thereafter.

## Discussion

The development of experimental infection models using competent tick vectors allowed for the assessment of acaricidal prophylactic treatments in preventing *B. canis* transmission under standardized laboratory conditions [12]. The importance of conducting such studies [11, 13, 15, 16, 21, 22], supporting any claims such as ‘aids in’ or ‘prevents the transmission of tick-borne pathogens’, has since been recognised by the latest WAAVP guidelines for evaluating parasiticides for the treatment, prevention and control of flea and tick infestations on dogs and cats, and has become an industry standard [23]. Although these challenge models allowed for the standardization of infection challenges within a trial, differences between studies with regard to design as well as *B. canis* infection rates in ticks have been observed. For example, in previous studies, infection rates of 8–25% were reported in experiments evaluating efficacy of systemic products [14–16, 22] and 2–44% in studies evaluating the efficacy of topically applied acaricides [11–13, 21]. In a recent review paper [3], the prevalence of *B. canis* in adult *D. reticulatus* ticks based on molecular screening of field collected ticks was reported. The prevalence rates ranged from 0% in surveys conducted for instance in Germany or Belarus to 0.7% in eastern Poland, 1.6% in the Netherlands, 2.3% in south-western Slovakia, 3.4% in Ukraine and 4.2% in Poland to as high as 14.7% in eastern Slovakia and 14.8% in southern Poland. In many instances the infection rate of ticks used in controlled studies were therefore much higher than that reported for ticks in the field. Since tick challenge models aim at simulating natural exposures in a standardised and controlled manner in the laboratory, the infection rates in ticks used for artificial challenges should also generally reflect those rates found in the field, at least at the higher spectrum of infection rates reported. Moreover, the tick infection rates used must ultimately result in infection rates in control dogs that validate the effectiveness of the challenge model, thereby simulating a realistic risk scenario likely to be encountered in the field. In the present study the dogs were challenged with ticks with an infection rate of 8% which was approximately in the middle of the infection rate range reported in the field (0.7–14.8%) [3].

In addition to natural tick infection rates, the natural host acquisition behaviour by ticks was also closely simulated. Since exposing dogs to ticks questing on vegetation was practically not feasible in a laboratory setup, it was opted to release the required number of ticks (*n* = 50) in an infestation crate, place the dog within the crate and expose the dog to the ticks for one hour. This method was regarded as the closest simulation possible for natural host acquisition behaviour by *D. reticulatus*. *In situ* tick counts performed during the study give an indication of the attachment and status (live or dead) of ticks on the dogs but they are not considered to be 100% accurate as some ticks might be missed (during removal tick counts dogs are also combed with a fine toothed flea comb to ensure accuracy of assessment). This may partially explain the low numbers of ticks found on control animals, with any ticks found on treated animals directly impacting any efficacy calculations. Because of the 48-hour minimal duration required for sporogony [24], *Babesia* spp. are generally considered as pathogens with slow transmission compared to other tick-borne pathogens. Consequently, recent models used to assess the efficacy of ectoparasiticides or repellents against transmission of *Babesia* spp. in dogs allow adult ticks to feed on the animals for at least four days [14]. Therefore, the acaricidal efficacy reported here is considered to be secondary. Using this approach in the present study, 26 out of 32 (81.25%) control dogs challenged with ticks were successfully infected based on the presence of *B. canis* DNA (PCR) and antibodies (IFA) with no infections observed in Seresto®–treated dogs. The challenge methodology used in this study was therefore regarded as highly effective.

The prevalence of infections with *Babesia* sp. reported from dogs in the field is considerably lower than that observed in the non-treated control dogs in the present study, possibly due to lower tick exposure found in field situations. For example, in a recent field investigation in Austria, Leschnik et al. [25] reported 3.3% of non-treated control dogs becoming infected with *B. canis* over a time period of 11 months. Therefore the experimental challenge model used in the present study can be regarded as a high risk scenario due to a much higher infection risk than that expected to occur naturally. The full protection of dogs (*n* = 8) treated with the Seresto® collar following monthly tick challenges up to eight months, can only be attributed to the sustained efficacy over this period which is in line with previously reported efficacy against *D. reticulatus* over this time period [20]. There may, however, be an attachment of single ticks. For this reason, a transmission of infectious diseases cannot be completely excluded if conditions are unfavourable. Moreover, to fully protect dogs against infection, the sustained efficacy against *D. reticulatus* had to induce mortality in all infected ticks before maturation of sporozoites in their salivary glands and subsequent infection of their canine host. The full protection of the Seresto®-treated dogs demonstrates that the rapid killing effect of Seresto® induced mortality in all infected ticks before maturation of sporozoites in their salivary glands and subsequent infection of their canine host. This result confirmed previous findings of full protection against *B. canis* infection after a single challenge with infected ticks one month after collar administration [11].

The result of the present study underlines the findings of other trials demonstrating the ability of the Seresto® collar to protect dogs against a variety of vector borne pathogens like *Anaplasma platys*, *B. vogeli*, *Ehrlichia canis* and *Leishmania infantum* [23, 26–28]. The long- term (eight months) efficacy of Seresto® therefore makes it an ideal product to protect dogs against *B. canis* infections as well as other vector borne diseases, and allows for the sustained protection of treated dogs throughout seasons of high risk.

## Conclusions

The Seresto® collar was highly effective against challenge with *D. reticulatus* ticks for eight months (entire duration of study period). The high sustained acaricidal efficacy over this period prevented *B. canis* transmission, thus fully protecting dogs against infection in this experimental infestation model.

## Abbreviations

GCP: Good clinical practice
IFA: Immunofluorescent assay
PCR: Polymerase chain reaction
